# Oligomer‐A*β*42 suppress glioma progression via potentiating phagocytosis of microglia

**DOI:** 10.1111/cns.14495

**Published:** 2023-10-17

**Authors:** Jie Lu, Zhenning Wang, Zhenqiang He, Yang Hu, Hao Duan, Zihao Liu, Depei Li, Sheng Zhong, Jiaoyan Ren, Guojun Zhao, Yonggao Mou, Maojin Yao

**Affiliations:** ^1^ Department of Neurosurgery/Neuro‐oncology Sun Yat‐sen University Cancer Center Guangzhou China; ^2^ State Key Laboratory of Oncology in South China, Collaborative Innovation Center for Cancer Medicine Sun Yat‐sen University Cancer Center Guangzhou China; ^3^ The First Affiliated Hospital of Guangzhou Medical University Guangzhou Institute of Respiratory Disease & China State Key Laboratory of Respiratory Disease Guangzhou China; ^4^ Department of Neurosurgery, Dongguan People's Hospital (Affiliated Dongguan Hospital) Southern Medical University Dongguan China; ^5^ School of Food Science and Engineering South China University of Technology Guangzhou China; ^6^ Laboratory Animal Center The Sixth Affiliated Hospital of Guangzhou Medical University Qingyuan China

**Keywords:** glioma, IGF‐1, microglia, OA*β*42, phagocytosis

## Abstract

**Aims:**

Glioma is characterized by an immunosuppressed environment and a poor prognosis. The accumulation of Amyloid *β* (A*β*) leads to an active environment during the early stages of Alzheimer's disease (AD). A*β* is also present in glioma tissues; however, the biological and translational implications of A*β* in glioma are elusive.

**Methods:**

Immunohistochemical (IHC) staining, Kaplan–Meier (KM) survival analysis and Cox regression analysis on a cohort of 79 patients from our institution were performed to investigate the association between A*β* and the malignancy of glioma. Subsequently, the potential of oligomer‐A*β*42 (OA*β*42) to inhibit glioma growth was investigated *in vivo* and *in vitro*. Immunofluorescence staining and phagocytosis assays were performed to evaluate the activation of microglia. Finally, RNA‐seq was utilized to identify the predominant signaling involved in this process and *in vitro* studies were performed to validate them.

**Results:**

A positive correlation between A*β* and a favorable prognosis was observed in glioma. Furthermore, OA*β*42 suppressed glioma growth by enhancing the phagocytic activity of microglia. Insulin‐like growth factor 1 (IGF‐1) secreted by OA*β*42‐activated microglia was essential in the engulfment process.

**Conclusion:**

Our study proved an anti‐glioma effect of A*β*, and microglia could serve as a cellular target for treating glioma with OA*β*42.

## Graphical abstract

In the early stages of Alzheimer's disease (AD), the activation of microglia by Oligomer‐A*β*42 (OA*β*42) results in a heightened phagocytosis function. The tumor associated microglia (TAMs) can be educated by OA*β*42 to unravel the masked phagocytosis function via up‐regulating the IGF‐1/IGF‐1R signaling.

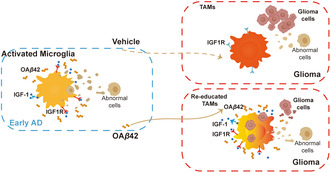

## 1 INTRODUCTION

Glioma is the most prevalent primary brain tumor, and its treatment has struggled for decades. Despite conventional chemotherapy, radiotherapy, and surgery, glioma's prognoses remain poor. Although the current immunotherapy achieved success in the peripheral system and have also been developed against glioma. Not surprisingly, current clinical trials of immunotherapies observed limited benefits for patients.^1^ Compared to the peripheral system, the brain's immune system has a very distinct structure, with microglia dominating while T cells are scarce.^2^ Microglia are the fundamental innate immune cells developmental, originating from the yolk sac within the brain.^3^ Numerous pieces of evidence have proved that microglia can mediate the immune surveillance of our brain by engulfing potential pathogens or mutant cells. Microglia also directly recognize, uptake, and degrade tumor cells through phagocytosis.^4^, ^5^ However, the primitive phagocytosis function of microglia was masked in the glioma environment.^6^, ^7^ Therefore, The efficient clearing of tumor cells by microglia is a promising strategy in glioma treatment.

The early stage of AD has an activated immune environment.^8^, ^9^ It is asymptomatic and distinct from the late stage with declined cognitive function. Moreover, the time course from the early stage to the late stage can be over 10 years.^10^ An inverse relationship between AD and cancer has been well‐established; that is, older people with AD had a lower cancer risk, and vice versa, which was reported by several epidemiological studies.^11^, ^12^, ^13^, ^14^, ^15^, ^16^ The representative work by Phillip P. Wolf from the heart center showed a 60% decreased cancer rate (including brain tumors) from AD patients.^11^ The observational evidence, the similar immune organism structure, and the status of microglia support the hypothesis that AD‐associated microglia are unfriendly to glioma.^17^ In this circumstance, the translational study based on the hypothesis can be valuable, and the immune agonist for the microglia activation could be essential.^18^

A*β*, the dominant metabolites of AD, accumulate in distinct phenotypes over the clinical stages of AD— the mono, oligomer, and fibrillar type with 40 or 42 amino acids of A*β*.^19^ Oligomer‐A*β* 42 (OA*β*42) can promote an inflammatory activation of microglia in the early stage of AD, representing the immune feature of the AD brain.^20^ Besides activating microglia in early AD, the A*β* can be regarded as a systemic peptide with many functions, including antibacterial, BBB protection, and Calcium homeostasis. The A*β* also associated with cancer. Previous study have revealed that rats with naked mole phenotypes never develop tumors and accumulate A*β* at levels similar to AD mice.^21^, ^22^ Another group proved that OA*β*42 could inhibit glioma growth via degenerating vascular capillaries.^23^ Indeed, A*β* has been demonstrated to exist in human and mouse glioma tissues.^24^, ^25^ However, the role of A*β* was not assessed in glioma, and whether OA*β*42 could be anti‐glioma needs to be evaluated thoroughly. In this study, we assessed the clinical role of OA*β*42 in clinical samples and public datasets. We also identified its potential application against glioma progression and the underlying mechanism.

## 2 MATERIALS AND METHODS

### 2.1 Patients

We retrospectively reviewed the medical documents of 79 patients diagnosed with glioma (except for pediatric subtypes) submitted to the Department of Neurosurgery, the Sun Yat‐sen University Cancer Center, from February 2011 to November 2017. The overall survival (OS) was defined as the period from the day of surgical operation to death or the latest follow‐up data updated on December 31, 2022. All clinical and pathological data were recorded, including the age and clinical diagnosis, histological pattern, and OS (Table S1). Univariate and multivariate Cox Regression Analyses were conducted to identify the glioma risk factor based on the OS. Kaplan–Meier (KM) survival analysis was performed to compare the OS outcomes of the A*β* positive and negative groups in the glioma cohort and GBM cohort from our center.

### 2.2 Public data analysis

An analysis of amyloid precursor protein (APP) and cleaved by *β*‐Site APP‐cleaving enzyme 1 (BACE1) expression in glioma tissue using GEPIA2 (http://gepia2.cancer‐pku.cn/) was carried out on 163 cases of glioblastoma multiforme (GBM) and 518 cases of low‐grade gliomas (LGGs).

### 2.3 Immunohistochemistry analysis

10% formalin‐fixed glioma tissues were embedded in paraffin and cut into sections. The immunohistochemistry staining was conducted using an immunostaining system (BenchMark ULTRA system, Ventana‐Roche, Switzerland) after deparaffinized, rehydrated, inactivated endogenous peroxidase, and antigen retrieval. The slides of tissue sections were treated with A*β*1‐42 antibody (at a concentration of 1:1000) and left to incubate overnight at a temperature of 4°C, then staining with 3,3′‐diaminobenzidine and counterstaining with hematoxylin, the specimen was dehydrated, xylene‐treated, and mounted. 3 peripheral glioma tissues from the 79 patients' cohort were applied as the normal control. 3 experienced neuropathologists examined and reviewed all slides, and representative pictures were captured with a Leica DM6 microscope (Leica).

### 2.4 Preparation of oligomeric amyloid *β*

We purchased human recombinant A*β*1‐42 (purity>95%) from Meilunbio, Dalian. Lyophilized peptide was diluted in 1,1,1,3,3,3‐hexafluoro‐2‐propanol (HFIP) 1 mg/mL at 4°C in order to decrease the sheet structures' formation and the helical structures' stable. The peptide was placed in a chemical fume hood to air dry for 1 h at room temperature, and further drying in a Speed Vac (Thermo‐Savant) for 30 min. A clear film was formed after resuspension in 100% dimethylsulfoxide (DMSO) at 1 mM, aliquoting, and storing at −80°C. To assemble the oligomers, the peptide film was resuspended in DMSO at 5 mM with water bash ultrasonic for 10 min, diluted to 100 mM in F‐12 medium (phenol red‐free) and incubated for 24 h at 4°C. Each sample was kept on ice and used in experiments the same day.

### 2.5 Atomic force microscopy (AFM)

To confirm the oligomeric state of the peptide, the AFM micrograph was taken immediately after dissolving A*β*42. The samples were tested with AFM (Dimension Edge) at Puchuan Company, Guangzhou. The mica surface was examined on 4 different regions to ensure similar structures were present. The images presented are subtracted from the top view containing height and error channel data (Figure S1A,B).

### 2.6 Cell culture

The murine glioma cell lines GL261 and the murine microglia cell line BV2 were obtained from the State Key Laboratory of Oncology in South China and used for this study. Mycoplasma infection was found to be absent in all cell lines tested. The GL261 cells and BV2 cells were maintained in DMEM with high glucose (Gibco BRL), 10% fetal bovine serum (FBS, HyClone Inc, Logan, UT), 100 U/mL penicillin and 100 μg/mL streptomycin, in a humidified atmosphere at 37 °C under 5% CO2. Supernatants were collected after glioma cells were cultured in an FBS‐free medium for 24 h, and then filtered with a polyethersulfone 0.22 μm membrane, serve as the glioma cell‐derived condition medium (GCM) for the usage of the *in vitro* experiments.

### 2.7 Establishment of the allogeneic glioma mouse model

Anesthesia was administered with isoflurane during all surgery, and all operations were made to minimize suffering. Mice aged 6–8 weeks received GL261 cells implanted into their right cerebral hemisphere. The implant was performed according to the description here. Incisions on the scalp were made in the midline after mice were anesthetized with isoflurane. The skull was drilled with a burr hole of 0.5 mm diameter at stereotaxic coordinates of the bregma, 2 mm lateral, 1 mm caudal, and 3 mm ventral. A 2 μL glioma cell suspension (1 × 10^5^ cells/μL in phosphate buffer solution (PBS)) was administered at a depth of 3 mm for 2 min. OA*β*42 (1 μM) treatment was treated in Glioma‐bearing mice by inoculating glioma cells. Moreover, the control group was inoculated with glioma and vehicle control (DMSO) with a corresponding concentration.

### 2.8 Analysis of mouse survival and brain tumor growth

Survival and glioma incidence of the mice were observed over 2 months after inoculating glioma cells and OA*β*42. In addition, mice were injected with D‐luciferin (Macklin, Shanghai, China; 100 μL intraperitoneally at 15 mg/mL in PBS) to image JX‐594‐driven luciferase expression post‐anesthetization. Data acquisition and analysis were performed using Indigo Image v2.0.5.0 software (Berthold). *In vivo* imaging was conducted with the NightOWL LB 983 *in vivo* Imaging System (Berthold) at every 7 days post the implantation.

### 2.9 Immunofluorescence staining

28 days post implantation, the glioma‐bearing mice were anesthetized with pentobarbital (50 mg/kg) and perfused with PBS followed by 4% paraformaldehyde (PFA) transcardially. Post‐fixed of the removed brain tissues in 4% PFA for 24 h, dehydrated by a followed sucrose at a concentration of 0.15 M, 0.5 M, and 0.8 M at 4°C. Using a Leica CM1950 cryostat, the brains were embedded in an optimal cutting‐temperature compound (Sakura). Staining of sections was performed according to our previous methods.^26^ Tissue sections were incubated with rabbit Ki‐67 primary antibody (CST, CA, 1:100), IBA1 primary antibody (Abcam, CA, 1:100), GFAP primary antibody from mouse(DAKO, CA, 1:100), CD3 primary antibody from rat(Biolegend, CA, dilution 1:100), and rabbit CD68 primary antibody (Biolegend, CA, 1:100) overnight at 4°C. Then, tissue sections were incubated with Alexa 488 or Alexa 555 conjugated anti‐mouse secondary antibodies, or Alexa 647 conjugated anti‐rabbit secondary antibodies for 60 min at room temperature. We examined tissue sections using a Zeiss LSM880 confocal microscope (Zeiss). Table S4 provides the antibodies used for this study.

### 2.10 The EdU assay

EdU was assayed using BeyoClick™ EdU Cell Proliferation Kit as directed by the manufacturer. First, the cells were exposed to the vehicle control (DMSO, 0.2%) and various concentrations of OA*β*42 for a duration of 24 h. Then, each well was treated with EdU (final concentration: 10 M) for 2 h at 37°C, after which the cells were harvested. Using Click Reaction Solution containing Azide 555 to mark EdU, 50 liters were added to each well, and the cells were incubated for 30 minutes at room temperature in the dark. Finally, cell nuclei were counterstained with Hoechst 33342 at room temperature for 10 min. The images were taken with a Leica DM6 microscope (Leica).

### 2.11 Live cell phagocytosis assay

Fluorescent beads phagocytosis assay was employed to analyze live microglia phagocytosis in co‐cultures with glioma cells. OA*β*42 in simple F12 medium was thawed on ice and polymerized in a 24‐well plate (Corning) at 37 °C. GL261 was labeled with GFP. BV2 was unstained and replicated by staining with CellTracke Red CMTPX (Yeasen). The plates were imaged for 10 h at the basetime of addition with lower concentration (1 μM), higher concentration (5 μM) OA*β*42, or vehicle in a motorized ImageXpress microscope (Molecular Devices). Image sequences were combined with the ImageXpress, and quantification was analyzed with ImageJ. Only when the GFP‐labeled cell interacts with the unstained cell a phagocytosis issue can be counted. And the phagocytosis index was identified as the reduced percentage of these GFP‐labeled cells from the base‐time to the ending time.

### 2.12 Latex bead phagocytosis assay

BV2 cells were seeded in 24‐well chambers (Corning) and treated with vehicle, lower concentration (1 μM), higher concentration (5 μM) OA*β*42 or IGFBP3 + 1 μM OA*β*42 for 12 h. Cells were captured after cultured with 1‐μm‐diameter latex beads conjugated to a red fluorescent protein (RFP) (Sigma) with a 10–20 beads per cell ratio. Alternatively, Green Actin Tracking Stain (Invitrogen) pretreated cells were filmed on coverslips, incubated with beads for 1 h, washed with PBS for three times, and treated with DMEM medium for an extra hour. Finally, cells in the chambers or on the coverslips were collected for immunofluorescence analysis. DNA was stained with DAPI (Life Technologies). 2 or more beads overlapping in the microglia localization can be determined as a phagocytosis issue. The phagocytosis index was identified as the ratio of phagocytosis in microglia. The images were captured by a DMi8 microscope (Leica) and a LSM880 confocal microscope (Zeiss).

### 2.13 Quantitative PCR (qPCR)

The steps were performed according to our previous methods.^26^ Briefly, total RNA was retrieved from BV‐2 cells grown after treated by OA*β*42 or vehicle with the EZ‐press RNA Purification Kit (EZBioscience) according to the manufacturer's instructions. Reverse transcription was conducted with an iScript™ cDNA synthesis kit (Bio‐Rad, Hercules). qPCR was conducted with a CFX96 PCR detection system (Bio‐Rad) using Bio‐Rad SYBRGreen® dye. A standard curve was established to calculate starting quantities by the iCycleriQ optical system software (Bio‐Rad). The experiments were performed in triplicate. The primer sequences applied in this study were provided in Table S5.

### 2.14 RNA extraction and sequencing

BV2 microglia cells pre‐trained with glioma condition medium and 1 μM OA*β*42 or vehicle were harvested. Total RNA was extracted with a RNAmini kit (Qiagen). Enrichment of mRNA, fragmentation, reverse transcription, and library construction were conducted with the Illumina Novaseq 6000, and data analysis were performed by Genergy Biotechnology Co. Ltd.

### 2.15 Western blot

Cell lysates were presented to SDS–polyacrylamide gel electrophoresis and then transferred to polyvinylidene difluoride membranes. Then, primary antibodies were prepared to incubate the blots at 4°C overnight. Next, the membrane was washed and incubated within a corresponded secondary antibody for 1 h at room temperature. At last, the protein signals were detected with an enhanced chemiluminescence. The antibodies prepared to detect the proteins of interest were also listed in Table S4.

### 2.16 Statistical analysis

Before data statistical analyses, the Shapiro–Wilk test was performed for the normality of data distribution, and Levene's test was performed for the assumption of homogeneity of variance. Only when the data reached a normal distribution and a homogeneity of variance would the unpaired Student's two‐tailed *t*‐test, one‐way ANOVA, or two‐way ANOVA be conducted. When the data did not reach a normal distribution and a homogeneity of variance, the non‐parametric equivalent, including the Mann–Whitney *U*‐Test and the Kruskal‐Wallis test, was conducted for analysis. To characterize the cohort, mean ± SD, median (interquartile range, IQR), and counts (percentages) were used to present normally distributed, non‐normally distributed, and categorical variables, respectively. The Cox‐proportional Hazards Regression was used to study the relationship between the variables and mortality. Only when a variable reaches *p* ≤ 0.1 in the univariate analysis will it be used in the multivariate analysis. In Cox regression analysis, KM survival curves were plotted for variables significantly associated with patient prognosis (*p* ≤ 0.05).

All statistical analyses in this study were calculated using the IBM SPSS 23.0 program and GraphPad Prism 8.

## 3 RESULTS

### 3.1 A*β*42 deposition is the prognosis biomarker in glioma

**FIGURE 1:**
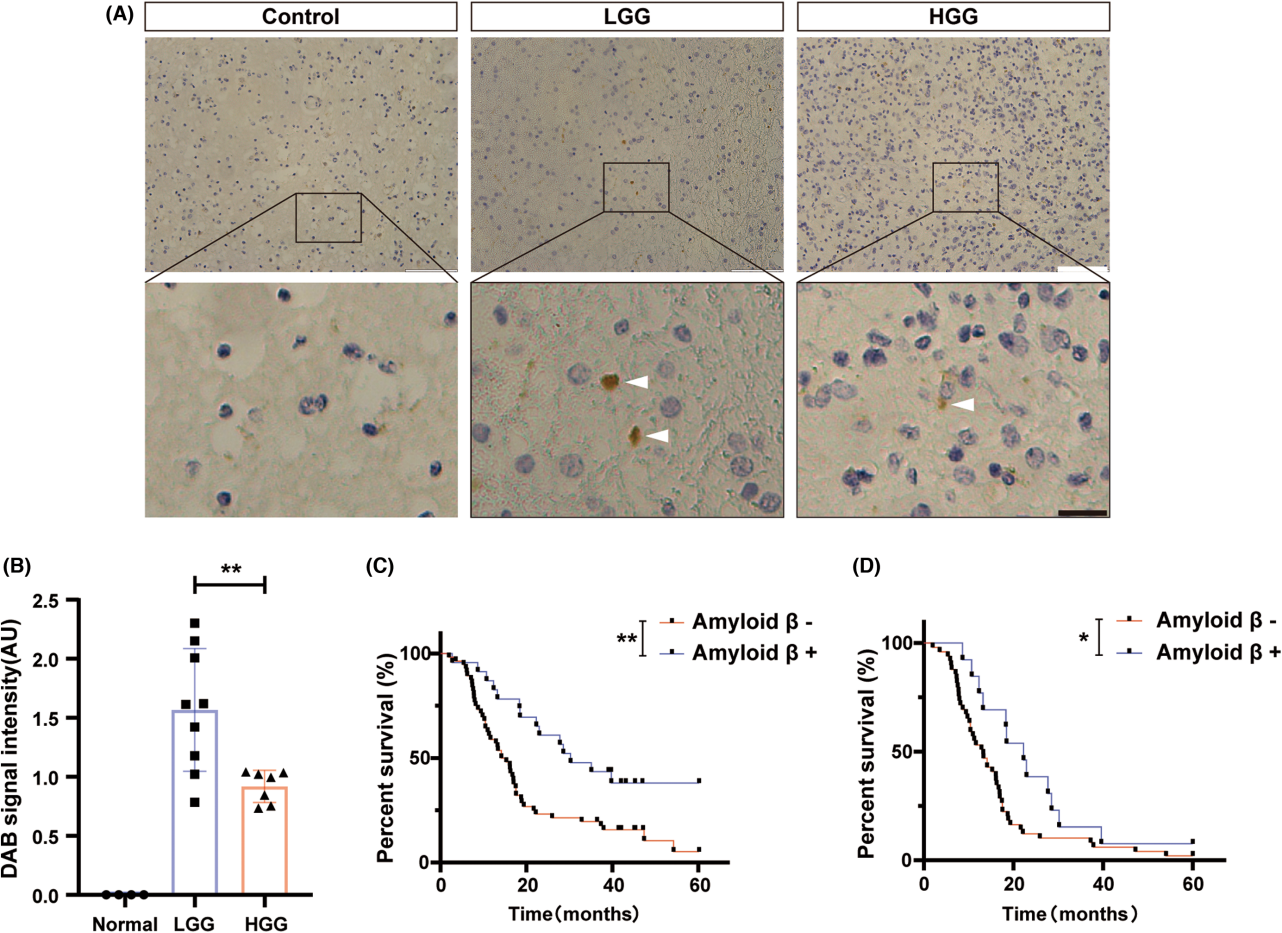
The clinical significance of A*β* in glioma patients. (A) Human glioma tissues were collected for IHC staining of A*β*. White bars represent 100 μm, and black bar represent 20 μm. Each grade of glioma tissues was repeated fourth, and the results were obtained and analyzed by experienced pathologists; (B) Quantification of DAB signal intensity was performed and represented in graphs, the non‐parametric equivalent with the Kruskal‐Wallis test was used; (C) Survival analysis of A*β* in 79 glioma patients' cohort, the Kaplan–Meier survival analysis with Log Rank test was applied. (D) Survival analysis of A*β* in 62 GBM patients' cohort, the Kaplan–Meier survival analysis with Log Rank test was conducted. Columns indicate median [95% CI]; n.s., not significant; *p* ≥ 0.05; *p < 0.05; **p < 0.01; ***p < 0.001.

To determine whether A*β* was associated with the malignancy of glioma, we performed IHC staining in tumor tissues from a cohort of 79 glioma patients, including LGG and HGG diagnosed in pathology, as well as 3 peripheral tumor tissues as the normal control (Table S1). A*β* with a higher expression level was examined in the LGG group compared to the HGG group (*p* = 0.0075; Figure 1A,B). To determine whether the A*β*‐positive could be an independent risk factor for glioma, univariate and multivariate Cox regression analysis was performed. A*β*‐positive was significantly related to long‐term OS (*p* = 0.029, *p* = 0.033; Tables S2 and S3). Furthermore, the OS of the A*β*‐positive group has a significantly improved OS compared to the negative group in the glioma and GBM cohort, respectively (*p* = 0.0025, *p* = 0.0391; Figure 1C,D).

To further confirm the clinical value of A*β*, we reviewed the production process for A*β*. A*β* was derived from amyloid precursor protein (APP) and cleaved by *β*‐Site APP‐cleaving enzyme 1 (BACE1). We enrolled TCGA database to analyze the correlation between APP or BACE1 and the OS of glioma patients. Though the level of the APP gene was not significantly different within the LGG and HGG patients, the high level of the BACE1 gene was positively correlated to the longer survival of glioma patients (*p* < 0.01; Figure S2A,B). The expression of the APP gene and the BACE1 gene was positively correlated in the glioma (*p* < 0.001; Figure S2C). These also supported the protective role of A*β* in glioma.

These results suggest that the expression of A*β* is negatively associated with glioma's malignant progression. Further exploration of how A*β* suppresses glioma growth is worthwhile.

### 3.2 OA*β*42 suppresses the progression of glioma *in vivo*

**FIGURE 2:**
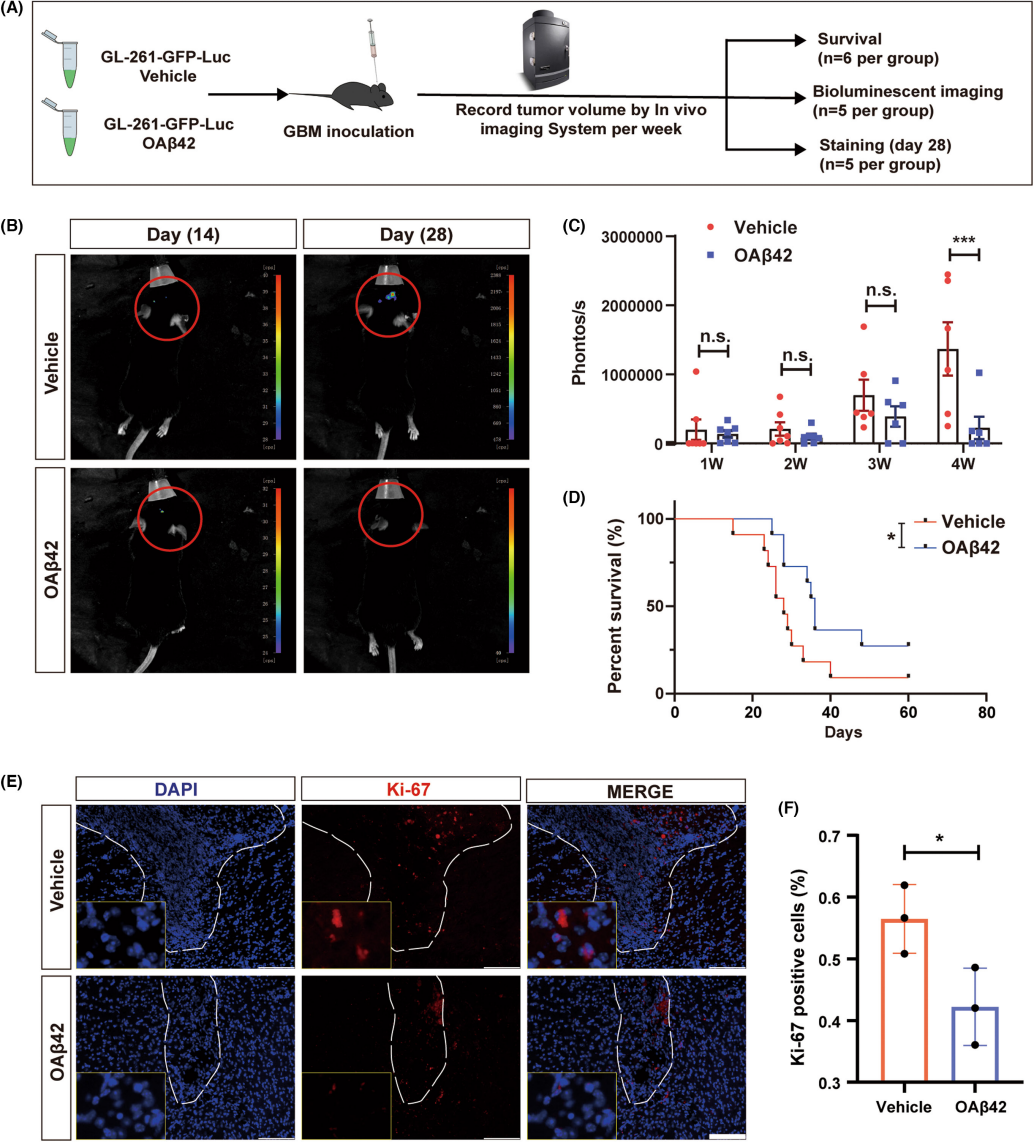
OA*β*42 suppresses glioma growth *in vivo*. (A) schematic diagram of OA*β*42 treatment in GL261‐inoculated C57 allogeneic model; (B) Representative (*In vivo* Imaging System) IVIS images of glioma‐inoculated mice treated with vehicle or OA*β*42 in early (2 weeks) and late stage (4 weeks); (C) Quantification of tumor volumes in two groups of mice recorded in IVIS, data was analyzed by the non‐parametric equivalent with Mann–Whitney *U*‐Test; (D) Survival analysis of glioma‐inoculated mice along with vehicle or OA*β*42 treatment, the Kaplan–Meier survival analysis with Breslow test was applied; (E, F) Representative images of Ki‐67 staining and quantification of Ki‐67 staining. White bars represent 100 μm, the Student's *t*‐test was conducted for the data analysis. Columns indicate median [95% CI]; n.s., not significant; *p* ≥ 0.05; *p < 0.05; **p < 0.01; ***p < 0.001.

To determine the potential role of A*β* in glioma formation, OA*β*42 (the dominant immune modulator) was prepared, and glioma cell line GL261 was inoculated in C57 mice. OA*β*42, or vehicle was co‐injected with the tumor cells simultaneously. Tumor volume was recorded by applying IVIS per week (Figure 2A). The tumor volume differentiation appeared in the 4th week after GL261 and OA*β*42 or vehicle inoculation (*p* = 0.0003; Figure 2B,C). After observing the mice and monitoring tumor volume per week, the prolonged OS was recorded and presented in the OA*β*42 treated group (*p* = 0.037; Figure 2D). Ki‐67 was stained in the two groups to assess the glioma's viability. The expression of Ki‐67 was decreased in the OA*β*42 group (*p* = 0.0421; Figure 2E,F). To further investigate the underlying mechanism of glioma inhibition induced by OA*β*42, microenvironment cells in the brain were considered.

### 3.3 OA*β*42 activates microglia in phagocytosis in the glioma microenvironment

**FIGURE 3:**
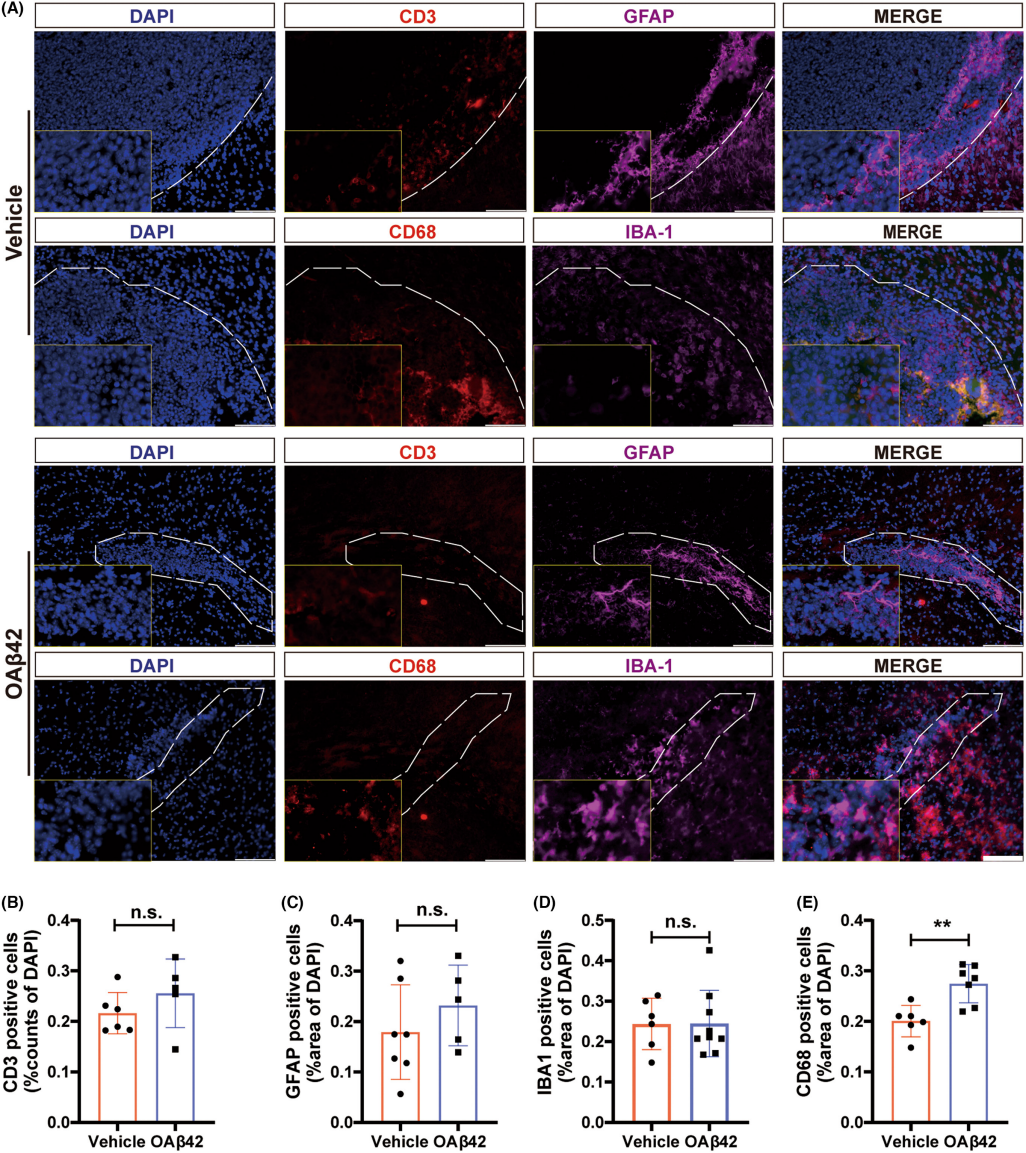
OA*β*42 active microglia and re‐modulate the tumor microenvironment. (A) Representative images of the immune microenvironment of Glioma‐bearing mice treated with vehicle or OA*β*42. White bars represent 100 μm; (B–E) Quantification of the immune cells in the two groups, the Student's *t*‐test was conducted for the data analysis. CD3 represented T cells (B), GFAP represented astrocytes (C), IBA1 represented microglia (D), and CD68 represented activated microglia (E). Columns indicate median [95% CI]; n.s., not significant; *p* ≥ 0.05; *p < 0.05; **p < 0.01; ***p < 0.001.

To detect changes in the microenvironment induced by OA*β*42 in glioma, IF staining with CD3, GFAP, IBA1, and CD68 was performed (Figure 3A). Both positive expressions of CD3, GFAP, and IBA1 showed no significant differences in glioma treated by OA*β*42 or vehicle (Figure 3B–D). However, the expression of CD68 was upregulated in the OA*β*42 group significantly (*p* = 0.003; Figure 3E). These revealed that the glioma microenvironment was relatively maintained in homeostasis even with the treatment of OA*β*42. In the meantime, microglia were activated in the treatment of OA*β*42. Therefore, the activation of microglia may be the cause of microenvironment homeostasis.

**FIGURE 4:**
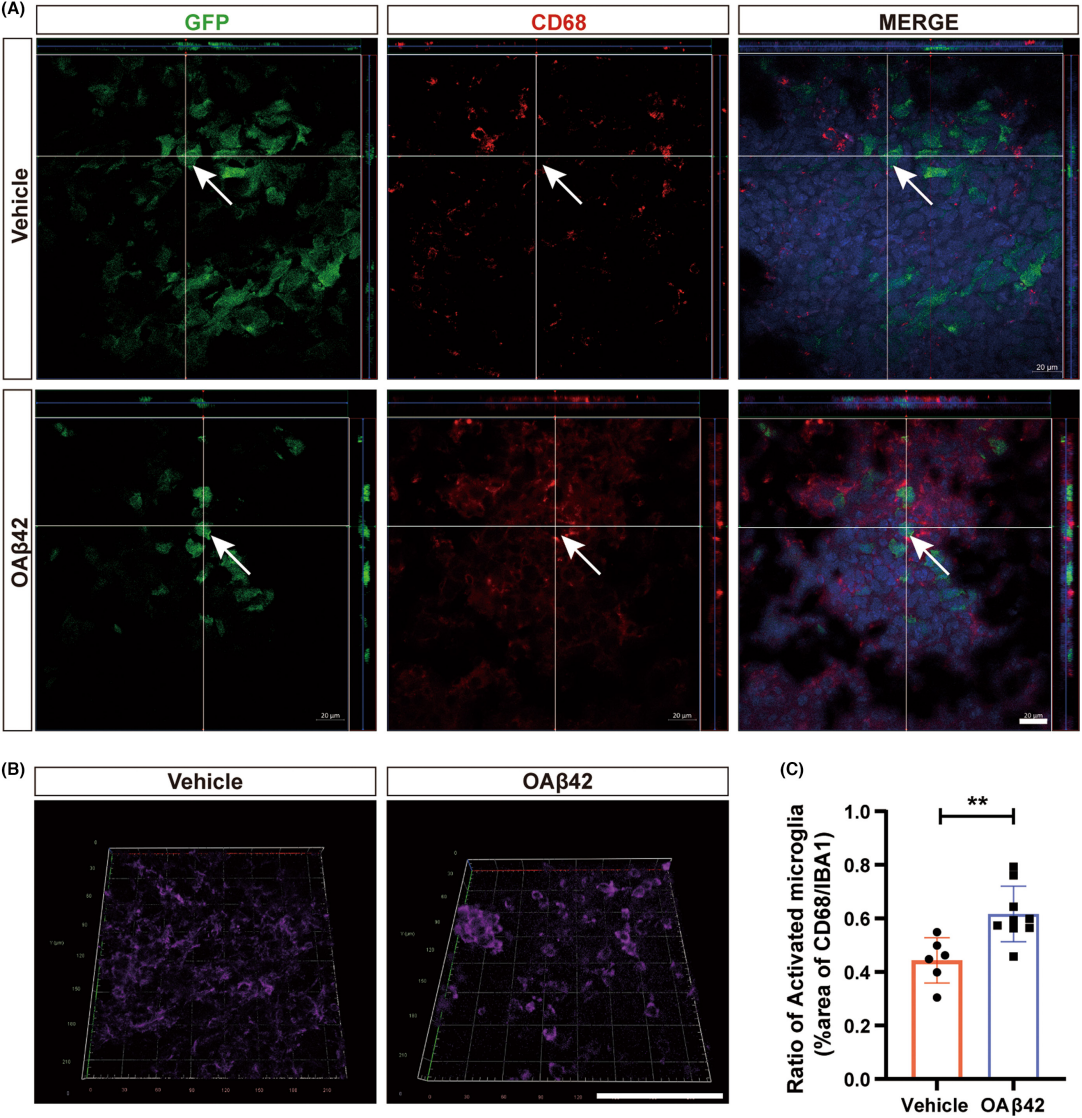
OA*β*42 potentiates phagocytosis of microglia. (A) Representative confocal images of microglia that engulf glioma cells after OA*β*42 stimulation, White bars represent 20 μm; (B) Representative images of microglia morphology with vehicle treatment or OA*β*42, White bars represent 100 μm; (C) Quantification of activation ratio by the ratio of CD68 to IBA1 positive microglia, the Student's *t*‐test was conducted for the data analysis. Columns indicate median [95% CI]; n.s., not significant; *p* ≥ 0.05; *p < 0.05; **p < 0.01; ***p < 0.001.

Meanwhile, we observed more phagocytosis phenomena in the OA*β*42 treated group. GFP‐labeled GL261 cells were surrounded by and close to the CD68‐positive microglia (Figure 4A). And ameboid phenotype of microglia appeared more frequently in the OA*β*42 treated group (Figure 4B). The high level of activated microglia in OA*β*42 was assured by the ratio of CD68 to IBA1 (*p* = 0.0049; Figure 4C).

**FIGURE 5:**
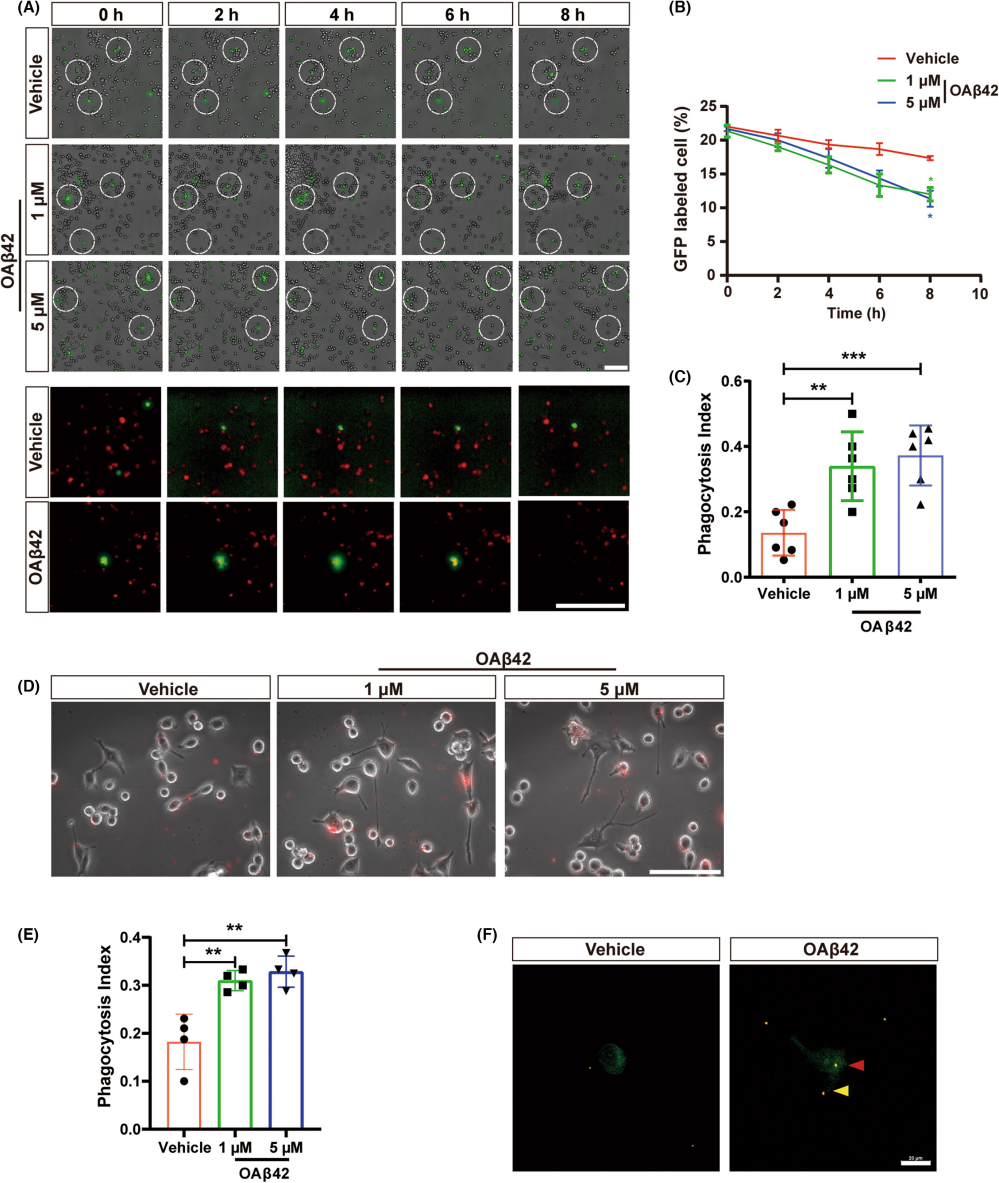
OA*β*42 enhances the phagocytosis capacity of microglia *in vitro*. (A) Dynamic process captured with ImageXpress microscope 10 h subsequent to the incubation of vehicle, low concentrate or high concentrate OA*β*42. GL261 cells were labeled with GFP and microglia without labeling at the top 3 panels. White dotted circles indicate the hotspots of cell interactions. 2 zoomed‐in panels of the co‐culture system containing GFP‐labeled GL261 cells and CellTracke Red CMTPX stained microglia were presented subsequently. White bars represent 100 μm; (B) Quantification for glioma cell inhibition in the co‐culture system, the two‐way ANOVA with Dunnett's multiple comparison test was conducted to data analysis. (C) Quantification for live cell phagocytosis assay in the co‐culture system, the one‐way ANOVA test with least significance difference post hoc comparison was performed to data analysis. (D) Representative images of microglia engulf beads subsequent to the incubation of vehicle, low concentrate, or high concentrate OA*β*42. White bars represent 100 μm; (E) Quantification of beads phagocytosis assay, the one‐way ANOVA test with least significance difference post hoc comparison was performed to data analysis; (F) Representative images of microglia engulfed beads with vehicle or OA*β*42 treatment. Microglia were labeled with CellMaskTM Green Actin Tracking Stain (1:1000) before beads were enrolled. The red arrow indicates the beads engulfed in the cell body, and the yellow arrow indicates the beads contacted by the cell processes. White bars represent 20 μm. Columns indicate median [95% CI]; n.s., not significant; *p* ≥ 0.05; *p < 0.05; **p < 0.01; ***p < 0.001.

To confirm the role of OA*β*42 in the anti‐glioma process, we performed direct cell culture and co‐culture experiments *in vitro*. The viability of glioma cells did not increase or decrease by OA*β*42 in a low concentration of OA*β*42 addition for 12 h (Figure S3A,B), consistent with the previous study by Paris et al.^23^ However, within the live cell observation station, under a suitable condition for cell incubation for 72 h, microglia and glioma cells were co‐cultured with vehicle or OA*β*42 for 10 h (Figure 5A). Glioma cell growth was inhibited in OA*β*42 exposure compared to vehicle exposure (Figure 5B). And the significant difference in glioma decrease emerged in the low or high concentrate OA*β*42 exposed groups at the 7th (n.s., *p* = 0.0219) and 8th (*p* = 0.0384, *p* = 0.048) hour, respectively. Phagocytosis issues, including contraction and overlap between cells, increased in the group of OA*β*42 addition. The increasing phagocytosis issues were induced under the low concentrate (*p* = 0.026) or high (*p* = 0.0007) concentrate OA*β*42 exposure (Figure 5C). The live glioma cell labeled with GFP engulfed by CellTracke Red CMTPX stained microglia was captured in the OA*β*42 group (Videos S1 and S2).

To study whether the ability to phagocytosis in microglia was enhanced by the single OA*β*42, a beads‐based phagocytosis assay was performed. Beads can be engulfed by microglia with OA*β*42 even at a low concentration (Figure 5D). The phagocytosis index showed a significant increase in the OA*β*42 of low (*p* = 0.0038) or high (*p* = 0.0015) concentration. Confocal microscopy was used to confirm phagocytosis and adherence to the surface (Figure 5F). The viability assay also ensured no toxicity of OA*β*42 to the microglia (Figure S3C,D).

These results indicate that the phagocytosis function in microglia was enhanced by OA*β*42 *in vitro*, which can explain the phagocytosis phenomenon observed *in vivo*. However, the potential molecular mechanisms remain unrevealed.

### 3.4 OA*β*42‐induced phagocytosis of microglia was mediated by up‐regulation of IGF‐1

**FIGURE 6:**
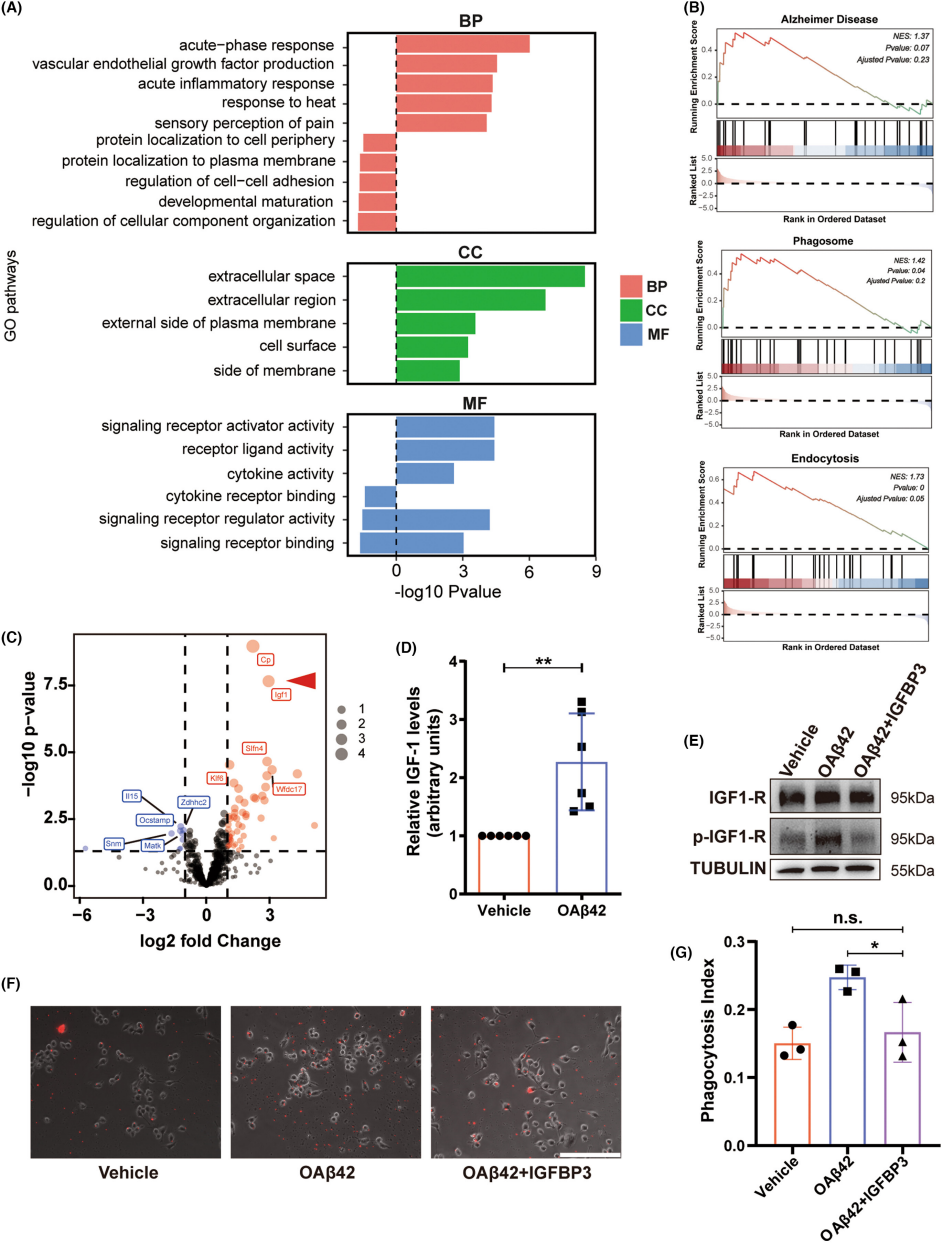
IGF1 is essential for the OA*β*42 induced microglia phagocytosis. (A) The Gene Ontology (GO) and functional pathway analysis of coding genes associated with positive correlation coefficients in TAMs treated with OA*β*42, *p*‐value ≤0.05. The x‐axis is the ‐log 10 *p* value; (B) GSEA of TAMs concerning genes upregulated in the exposure of OA*β*42. The analysis demonstrates a positive correlation (*q* < 0.1, Enrichment Score = 0.15) between many genes upregulated in phagocytosis and AD; (C) PCA of the transcriptome of glioma condition medium pre‐treated BV2 (TAMs) treated with OA*β*42 compared to vehicle. Volcano plot shows gene expression differences between microglia treated with OA*β*42 and vehicle. Log 2 gene ratios are plotted against negative log 10 *p* values; (D) qPCR reveals IGF‐1 upregulated in OA*β*42 stimulated microglia, the non‐parametric equivalent with Mann–Whitney *U*‐Test was performed to data analysis; (E) Western blot of IGF‐1 signaling related proteins of microglia treated with vehicle, OA*β*42, and OA*β*42+ IGFBP3; (F) IGFBP3 decreased the phagocytosis of microglia treated with OA*β*42. White bars represent 100 μm; (G) Quantification of the phagocytosis assay, the one‐way ANOVA test with least significance difference post hoc comparison was performed to data analysis. Columns indicate median [95% CI]; n.s., not significant; *p* ≥ 0.05; *p < 0.05; **p < 0.01; ***p < 0.001.

To find out the molecular mechanism of enhanced phagocytosis of microglia stimulated by OA*β*42, we compared expression profiles among BV2 + vehicle, BV2+ OA*β*42, TAMs+vehicle, and TAMs+ OA*β*42. To obtain a biological understanding of these OA*β*42‐phagocytosis relationships, enrichment analysis was conducted on each module significantly associated with GO terms. Upregulated cell surface receptor signaling pathway, external side of the plasma membrane, and cell adhesion molecule binding were the relevant biological process, molecular function, and cellular component, respectively (Figure 6A and Figure S4B). Consequently, molecular pathways associated with phagocytosis and AD were assessed. To find out the related biological pathways, gene set enrichment analysis (GSEA) was performed. Pathways related to regulated mononuclear cell migration, signal receptor binding, and wound healing were enriched among positive genes (Figure S4C–E), supporting the changes in phagocytosis partially characterize the function of OA*β*42. Furthermore, the AD, phagosome, and endocytosis pathways were enriched among positive gene loadings and glioma condition medium pretreatment and OA*β*42 (Figure 6B). Principal component analysis (PCA) revealed that IGF‐1 was the common top gene enriched in the OA*β*42 stimulated microglia and the glioma condition medium pretreated microglia (Figure 6C and Figure S4A). Together, these data provide differences in global expression and specific pathways in all samples, indicating that IGF‐1 was molecularly expressed in the phagocytosis state of OA*β*42 treated microglia.

To ensure the role of IGF‐1 in phagocytosis, we obtained the gene expression of IGF‐1 by qPCR (Figure 6D). A significant upregulation of IGF‐1 was detected (*p* = 0.0039). To make sure whether the IGF‐1 related receptor can be activated in the OA*β*42‐treated microglia phagocytosis, the Anti‐Phospho‐IGF‐I receptor was examined by western blot. The relatively high level of Phospho‐IGF‐I receptor appeared in the single OA*β*42 group, indicating that IGF‐1 activated the phagocytosis microglia indeed (Figure 6E). Furthermore, IGFBP3 neutralized the IGF‐1 induced by OA*β*42 *in vitro*. A significant decrease in the phagocytosis index of the IGFBP3+ OA*β*42 group was quantified compared to the single OA*β*42 group (*p* = 0.0409; Figure 6F,G). This indicates that the IGF‐1 secreted by microglia showed an enhanced phagocytosis function for the microglia itself indeed.

## 4 DISCUSSION

The immune microenvironment plays a critical role in the pathological development of AD. Microglia are the most dominant myeloid‐derived immune cells in the brain. Microglia activation has been identified as a prominent feature of the AD microenvironment and could drive AD's pathological process. In the tumor ecosystem, tumor cells were surrounded by various immune cells that could act in pro‐ and anti‐tumorigenic functions.^27^ Increasing evidence showed that higher densities of tumor‐associated microglia (TAMs) were found in HGG compared with LGG, suggesting TAMs serve as a villain in the environment.^28^, ^29^ TAMs can be divided into brain‐resident macrophages (microglia) and bone marrow‐derived macrophages (BMDMs) based on developmental origin.^30^ Microglia developed from precursors of erythromyeloids in the yolk sac and spontaneously regenerated during homeostasis.^31^, ^32^, ^33^ Moreover, microglia are also resistant to myeloablative irradiation with genetic stability.^34^, ^35^ Only in the cases of blood–brain barrier (BBB) disruption, such as glioma progression, could BMDMs be recruited into the brain and become TAMs.^36^ The primitive functions of microglia are maintaining the homeostasis of the brain through phagocytosis and the scavenging of abnormal cells or debris. In the current study, we attempted to reeducate the TAMs to normal status with the restored engulfing function to delay the glioma progression. We found that OA*β*42, the immune stimulator of early AD, can enhance microglia phagocytosis of glioma cells.^37^ These results indicated that the intrinsic function of microglia was masked in the tumor environment and can be resettled by niche modulators such as OA*β*42. And the results were also consistent with the observations that lower glioma incidence found in the AD population.

Sporadic attempts targeting microglia to treat glioma were reported. Colony stimulating factor 1‐receptor (CSF1‐R) is predominantly expressed by microglia and necessary for its survival.^38^ Blocking CSF1‐R in glioma‐bearing mice led to a reduced infiltration of GAMs and decreased tumor volume significantly.^39^ However, the CSF1‐R inhibitors failed in clinical trials.^40^ The fundamental cause of the failure lies in the mechanism that the CSF1‐R inhibitor modified the pro‐inflammation of microglia but failed to re‐educate the microglia into a normal state with healthy functions. Another research found the CD47/SIRPα axis as a “do not eat me signal” of glioma and blocking CD47 effectively restraining the glioma progression.^41^, ^42^ But severe hemolytic reaction caused by CD47 monoclonal antibody was noted in clinical trials.^43^ Though the anti‐CD47 therapy did not concern the re‐education of microglia and failed in the pre‐clinical stage, the enhanced phagocytosis was proved an effective target in glioma treatment. While both CSF1‐R and CD47/SIRPα are vital for microglia to survive in the physical status, the effectiveness of targeting these targets was limited in a pathological status. Our results provide a new angle in drug development against glioma, based on the pathological character of the target cells and exert its primitive functions. Therefore, re‐educating microglia based on their plasticity of phagocytosis, rather than clearance themselves, can serve as a promising strategy in glioma therapy.

Phagocytosis can be modulated by cytokines, adhesion to the matrix, as well as by host colony‐stimulating factors and microbial stimuli.^44^ Limited evidence is known about the role of growth factors in the phagocytosis of innate immune cells. IGF‐1, as a growth hormone, is conventionally known to stimulate cancer cell proliferation and tumor growth.^45^, ^46^ Indeed, IGF‐1 possesses pleiotropic properties that directly stimulate monocyte and macrophage to regulate inflammatory responses via enhanced TNF alpha production and chemotactic migration.^47^, ^48^ Recent studies reported that IGF‐1 could regulate the phagocytosis of astrocytes and epithelial cells.^49^, ^50^ Here, our study demonstrated that IGF‐1 is the essential factor in the phagocytosis of microglia enhanced by OA*β*42, helping to inhibit glioma growth. Two possibilities may explain the diverse function of IGF‐1: 1. The plasticity or status of macrophages stimulated by OA*β*42 in the brain differs from that caused by inflammatory factors of bronchial injury in the lung; 2. Though the lung‐resident macrophage and the brain‐resident microglia originated from the yolk sac, they live within a unique organ environment. These two resident engulfing cells acquired tissue‐specific adaptability within the terminated differentiation organ and reacted in distinct cellular reactions to scavenge abnormal cells or debris. In cellular endocytosis, IGF‐1 signaling is essential for mitochondrial biogenesis, mitophagy, and the Golgi apparatus.^51^ And the clearance of A*β* can be efficient for microglia in a sustained condition. Moreover, lower serum IGF‐1 level was observed to increase the risk of AD incidents.^52^, ^53^ These all indicated that IGF‐1 could benefit glioma phagocytosis by microglia. Besides the benefits of microglia phagocytosis, the pro‐function of glioma cells of IGF‐1 should also be considered. As the glioma cells were not proliferated *in vivo* and *in vitro* co‐culture systems, the concentration of IGF‐1 secreted by microglia might be low enough and affect microglia itself locally.

Other than IGF‐1, another concern in our study may lie in the side effect of OA*β*42. Although the consensus for the causative role of A*β* is uncertain, as we mentioned in the background, A*β* is a representative biomarker for AD. Indeed, the amyloid hypothesis is one of the speculations for the etiology of AD.^54^ The A*β* targeted peptide could not inhibit AD progression.^55^ The concentration of A*β* in cerebrospinal fluid was changed 10 years before the occurrence of AD.^56^ A*β* is more like a systemic metabolic peptide with no toxicity in a proper phenotype and limited concentration. Oligomer and 42 amino acid of A*β* is the representative phenotype of the immune modulation in the early stage of AD. The concentration of OA*β*42 was referred to in the previous study, and our cell proliferation experiment demonstrated that a low concentration of OA*β*42 was not toxic to the astrocytes and microglia. Thus, applying the OA*β*42 in low concentration can be safe. Though a more extended observation of the behavior in cognition and motivation could be strong evidence for the safety of OA*β*42, the vehicle group could not survive to the more extended destination. The future direction of the application of OA*β*42 could be medicated locally, and the developed A*β* antibodies could clear out the peptides.

Our study demonstrated that the anti‐glioma role of A*β* and the OA*β*42 could inhibit glioma growth via potentiating microglia phagocytosis. Furthermore, the microglia could be the target cell with elevated IGF‐1/IGF‐1R signaling in the glioma treatment.

## CONFLICT OF INTEREST STATEMENT

The authors have no conflict of interest.

## Supporting information


Appendix S1.

Video S1.

Video S2.

## Data Availability

The GEPIA2 Data is available in public (http://gepia2.cancer‐pku.cn/). The datasets generated during the current study are available from the corresponding author on reasonable request.
