# Supplementary material for: Oligomer‐Aβ42 suppress glioma progression via potentiating phagocytosis of microglia
Source: CNS Neurosci Ther. 2023 Oct 17;30(1):e14495. doi: 10.1111/cns.14495 (PMC10805446; doi:10.1111/cns.14495)
Supplement: Supplementary file 1 — Appendix S1. [file CNS-30-e14495-s003.doc]

**Title Page:**

**Oligomer-A*β*42 Suppress Glioma Progression via Potentiating Phagocytosis of Microglia**

**Running Title:**

**An Advanced Phagocytosis Glioma Therapy: Insight from AD Therapy**

Jie Lu^1,2,4^*, Zhenning Wang^3^*, Zhenqiang He^1,2^, Yang Hu^4^, Hao Duan^1,2^, Zihao Liu^4^, Depei Li^1,2^, Sheng Zhong^1,2^, Jiaoyan Ren^5^, Guojun Zhao^6^#, Yonggao Mou^1,2^#, Maojin Yao^4^#

1 Department of Neurosurgery/Neuro-oncology, Sun Yat-sen University Cancer Center, Guangzhou 510060, P. R. China.

2 State Key Laboratory of Oncology in South China, Collaborative Innovation Center for Cancer Medicine, Sun Yat-sen University Cancer Center, Guangzhou 510060, P. R. China.

3 Department of Neurosurgery, Dongguan People's Hospital (Affiliated Dongguan Hospital, Southern Medical University), Dongguan 523058, P. R. China.

4 The First Affiliated Hospital of Guangzhou Medical University, Guangzhou Institute of Respiratory Disease & China State Key Laboratory of Respiratory Disease, Guangzhou, 510182, China.

5 School of Food Science and Engineering, South China University of Technology, Guangzhou 510640, China.

6 Laboratory Animal Center, the Sixth Affiliated Hospital of Guangzhou Medical University, Qingyuan 511518, P. R. China.

* These authors have contributed equally to this work.

# Correspondence authors:

Guojun Zhao

Address: The Sixth Affiliated Hospital of Guangzhou Medical University, Qingyuan City People's Hospital, B24 Yinquan Road, Qingyuan 511518, Guangdong, China.

E-mail: zhaoguojun@gzhmu.edu.cn

Yonggao Mou,

Address: Department of Neurosurgery/Neuro-oncology, Sun Yat-sen University Cancer Center, 651 Dongfeng Road East, Guangzhou 510060, China.

E-mail: mouyg@sysucc.org.cn

Maojin Yao,

Address: The First Affiliated Hospital of Guangzhou Medical University, Guangzhou Institute of Respiratory Disease & China State Key Laboratory of Respiratory Disease, 195 Dongfeng Road West, Guangzhou 510182, China.

E-mail: [yaomaojin@gird.cn](mailto:yaomaojin@gird.cn)

Tel: +86 18373317133

**
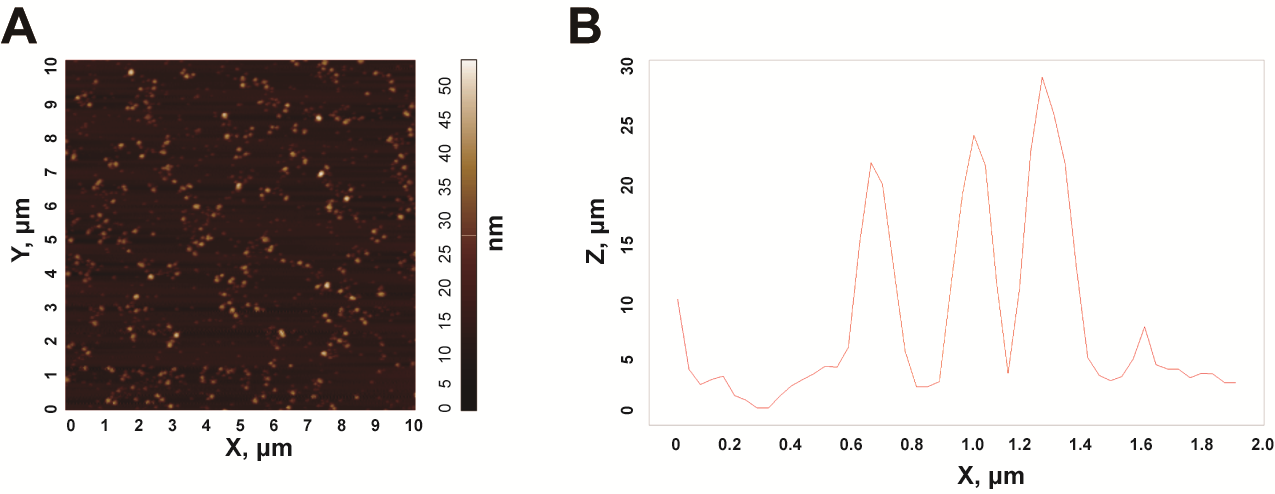
**

**Figure S1** Oligomer A*β*42 prepared for experiments. (A) The 10 μm × 10 μm field of the random field was captured with the tapping mode AFM of freshly prepared A*β* oligomers. The height and rough shape of the particle were qualitatively observed according to its lightness and darkness of color. (B) The height-line segment position curve with a length of 1.95 μm was randomly selected, it can be estimated that the average height of the particles is about 34 nm, and the average diameter is about 0.30 μm (the average value of three particles).


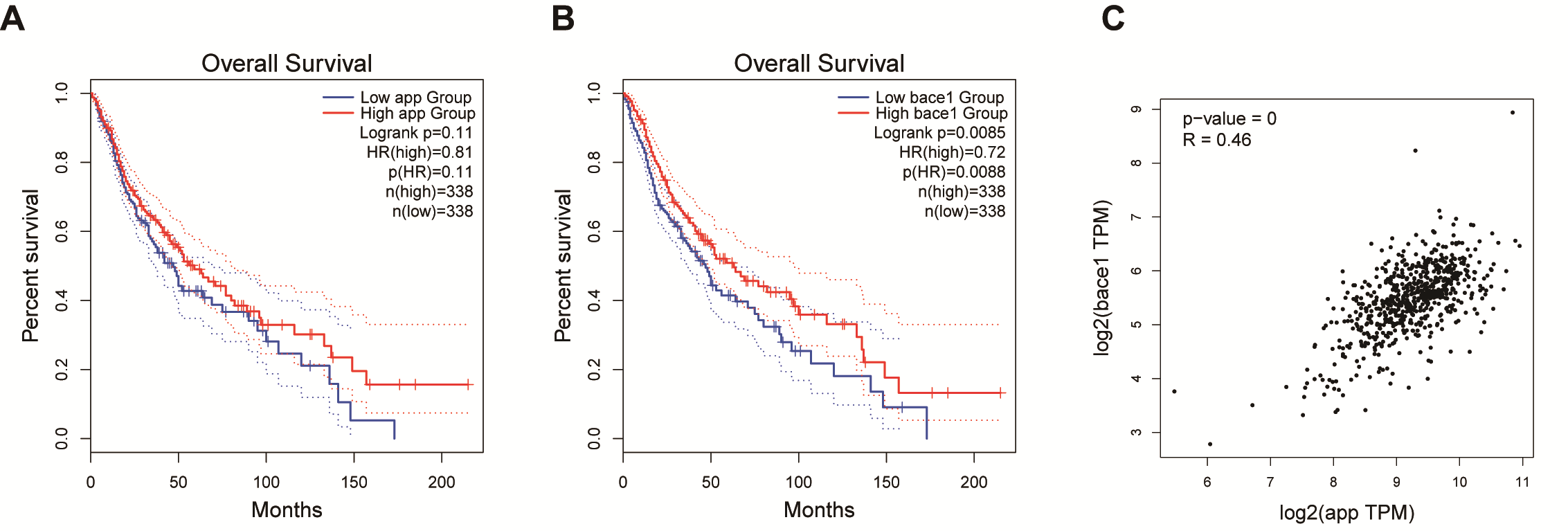


**Figure S2** Evaluation of the prognostic value of A*β* production-related genes in GEPIA2 datasets. (A) APP expression had no significant differentiation in the median OS of patients with glioma. (B) Patients with high BACE1 expression groups had a longer median OS than low BACE1 expression groups. (C) APP expression was positively correlated with BACE1 expression in glioma.

**
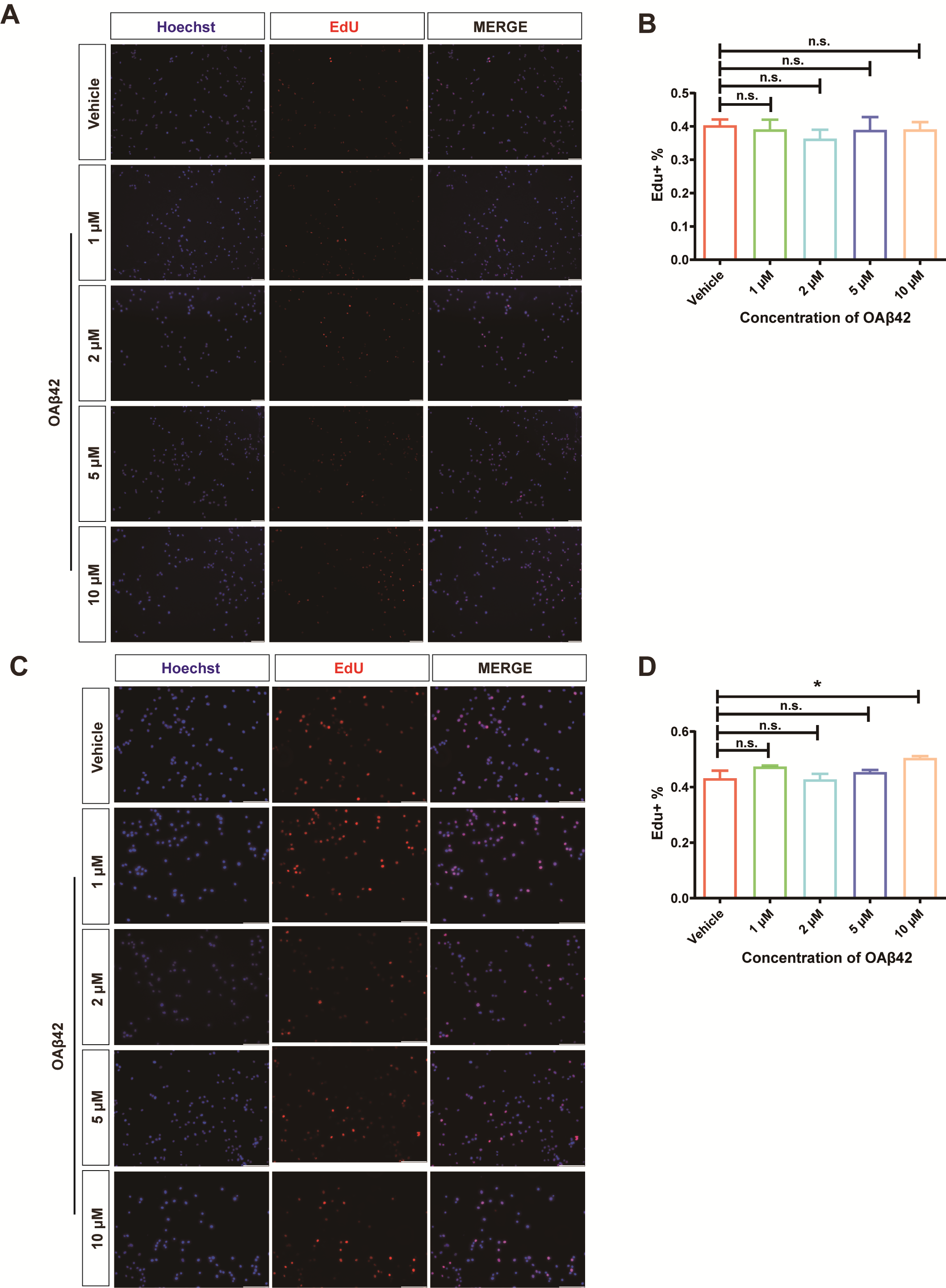
**

**Figure S3** Low concentration of OA*β*42 does not affect the viability of microglia and glioma cells. (A) Representative images of glioma cells stained with EdU (red). Nuclei were stained with Hoechst (blue). Cells treated with vehicle or OA*β*42 in increasing concentration (1 μM, 2 μM, 5 μM, 10 μM). (B) The number of proliferating glioma cells (EdU+ cells) was counted and expressed as a percentage of total microglia. (C) Representative images of microglia stained with EdU (red). Nuclei were stained with Hoechst (blue). Cells treated with vehicle or OA*β*42 in increasing concentration (1 μM, 2 μM, 5 μM, 10 μM). (D) The number of proliferating microglia (EdU+ cells) was counted and expressed as a percentage of total microglia. Columns indicate median [95% CI]; n.s., not significant; *P* ≥ 0.05; **P* < 0.05; ***P* < 0.01; ****P* < 0.001.


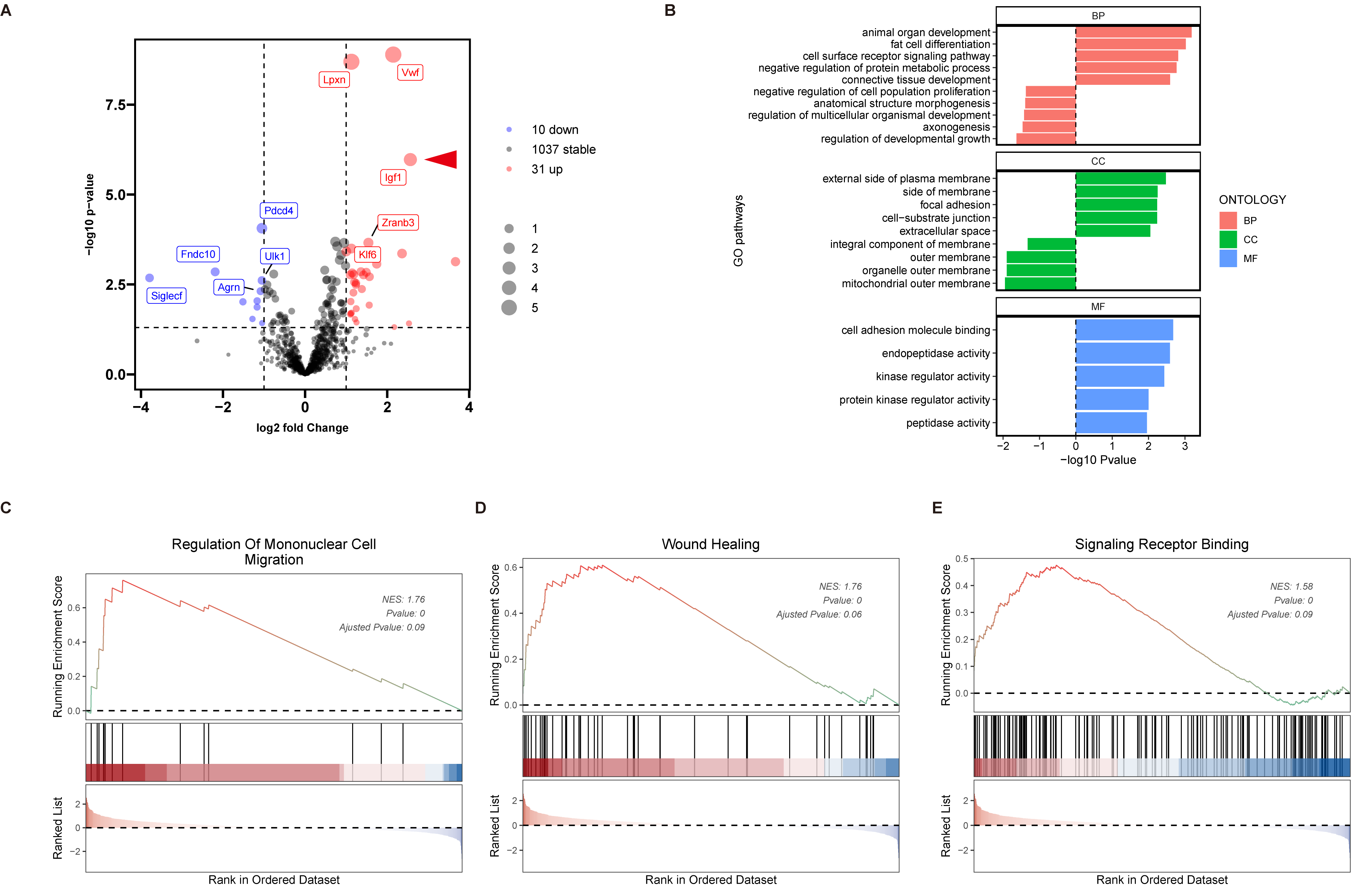


**Figure S4** IGF1 was identified as the difference expressed gene of the OA*β*42 induced microglia. IGF-1 level elevated in OA*β*42 induced microglia. (A) PCA of BV2 treated with OA*β*42 compared to vehicle. Volcano plot shows gene expression differences between microglia treated with OA*β*42 and vehicle. Log 2 gene ratios are plotted against negative log 10 *P* values; (B) GO analysis and pathway analysis of coding genes associated with positive correlation coefficients in BV2, *P*-value ≤ 0.05. The x-axis is the -log10 *P* value; (C–E) GSEA of microglia concerning genes upregulated in the exposure of OA*β*42. The analysis demonstrates a significant correlation (q < 0.1, Enrichment Score = 0.15) between many genes upregulated in the signaling of microglia phagocytosis.

**Table S1 Clinical data and histological classification**

| **Characteristics** | **Classification** | **Patients (n=79)** | **Aβ- (n=55)** | **Aβ+ (n=24)** | ***p*-value** |
| --- | --- | --- | --- | --- | --- |
| Age | ≥45y | 32 (40.5%) | 22 (40.0%) | 10 (41.7%) | 1.000 |
|  | <45y | 47 (59.5%) | 33 (60.0%) | 14 (58.3%) |  |
| Gender | Male | 49 (62.0%) | 35 (63.6%) | 14 (58.3%) | 0.846 |
|  | Female | 30 (38.0%) | 20 (36.4%) | 10 (41.7%) |  |
| Size | ≥50mm | 34 (43.0%) | 22 (40.0%) | 12 (50.0%) | 0.563 |
|  | <50mm | 45 (57.0%) | 33 (60.0%) | 12 (50.0%) |  |
| Single lesion | Yes | 71 (89.9%) | 51 (92.7%) | 20 (83.3%) | 0.386 |
|  | No | 8 (10.1%) | 4 (7.3%) | 4 (16.7%) |  |
| Brain-zone | Supratentorial | 72 (91.1%) | 50 (90.9%) | 22 (91.7%) | 0.164 |
|  | Subtentorial | 3 (3.8%) | 1 (1.8%) | 2 (8.3%) |  |
|  | Midline/Brainstem | 4 (5.1%) | 4 (7.3%) | 0 (0.0%) |  |
| Ventricle oppressed | Yes | 47 (59.5%) | 31 (56.4%) | 16 (66.7%) | 0.543 |
|  | No | 32 (40.5%) | 24 (43.6%) | 8 (33.3%) |  |
| En bloc resection | Yes | 56 (70.9%) | 38 (69.1%) | 18 (75.0%) | 0.793 |
|  | No | 23 (29.1%) | 17 (30.9%) | 6 (25.0%) |  |
| Ventricle opening | Yes | 55 (69.6%) | 35 (63.6%) | 20 (83.3%) | 0.138 |
|  | No | 24 (30.4%) | 20 (36.4%) | 4 (16.7%) |  |
| Relapse | Yes | 54 (68.4%) | 43 (78.2%) | 11 (45.8%) | 0.01 |
|  | No | 25 (31.6%) | 12 (21.8%) | 13 (54.2%) |  |
| KPS | >70 | 58 (73.4%) | 39 (70.9%) | 19 (79.2%) | 0.626 |
|  | ≤70 | 21 (26.6%) | 16 (29.1%) | 5 (20.8%) |  |
| Pathological subtypes | A | 4 (5.1%) | 1 (1.8%) | 3 (12.5%) | 0.149 |
|  | AA | 5 (6.3%) | 3 (5.5%) | 2 (8.3%) |  |
|  | AO | 4 (5.1%) | 2 (3.6%) | 2 (8.3%) |  |
|  | AE | 1 (1.3%) | 0 (0.0%) | 1 (4.2%) |  |
|  | O | 3 (3.8%) | 2 (3.6%) | 1 (4.2%) |  |
|  | GBM | 62 (78.5%) | 47 (85.5%) | 15 (62.5%) |  |
| WHO Classification | Ⅱ | 7 (8.9%) | 3 (5.5%) | 4 (16.7%) | 0.07 |
|  | Ⅲ | 10 (12.7%) | 5 (9.1%) | 5 (20.8%) |  |
|  | Ⅳ | 62 (78.5%) | 47 (85.5%) | 15 (62.5%) |  |
| Clinical Classification | LGG | 17 (21.5%) | 8 (14.5%) | 9 (37.5%) | 0.047 |
|  | HGG | 62 (78.5%) | 47 (85.5%) | 15 (62.5%) |  |
| Radio-Chemo therapy | No | 22 (27.8%) | 17 (30.9%) | 5 (20.8%) | 0.48 |
|  | Yes | 48 (60.8%) | 31 (56.4%) | 17 (70.8%) |  |
|  | Single | 9 (11.4%) | 7 (12.7%) | 2 (8.3%) |  |
| IDH mutation | Positive | 22 (27.8%) | 14 (25.5%) | 8 (33.3%) | 0.656 |
|  | Negative | 57 (72.2%) | 41 (74.5%) | 16 (66.7%) |  |
| MGMT methylation | Positive | 56 (70.9%) | 38 (69.1%) | 18 (75.0%) | 0.793 |
|  | Negative | 23 (29.1%) | 17 (30.9%) | 6 (25.0%) |  |
| 1P/19Q | No deletion | 54 (68.4%) | 39 (70.9%) | 15 (62.5%) | 0.132 |
|  | Co-deletion | 6 (7.6%) | 2 (3.6%) | 4 (16.7%) |  |
|  | Single | 19 (24.1%) | 14 (25.5%) | 5 (20.8%) |  |
| Ki67 | ≥30% | 51 (64.6%) | 38 (69.1%) | 13 (54.2%) | 0.308 |
|  | <30% | 28 (35.4%) | 17 (30.9%) | 11 (45.8%) |  |

Number (n); Ventricle opening (Procedure in the tumor resection) Pathological pattern (A astrocytoma, O oligodendroglioma, AO anaplastic oligodendroglioma, AA anaplastic astrocytoma, AE anaplastic ependymoma, GBM glioblastoma); LGG (Lower-grade glioma, including WHO grade Ⅱ, Ⅲ); HGG (High-grade glioma, including WHO grade Ⅳ); A*β* status (- = negative stained, + = positive stained).

**Table S2 Univariate Cox Regression analysis of risk factors for Overall Survival based on Survival status**

| **Characteristics** | **Classification** | **Hazard ratio (95% CI)** | ***p*-value** |
| --- | --- | --- | --- |
| Age | ≥45y | Ref |  |
|  | <45y | 2.44(1.458, 4.083) | 0.001 |
| Gender | Male | Ref |  |
|  | Female | 0.57(0.337, 0.964) | 0.036 |
| Size | ≥50mm | Ref |  |
|  | <50mm | 0.778(0.479, 1.264) | 0.311 |
| Single lesion | Yes | Ref |  |
|  | No | 1.108(0.525, 2.342) | 0.787 |
| Brain-zone | Supratentorial | Ref |  |
|  | Subtentorial | 0.486(0.117, 2.015) | 0.32 |
|  | Midline/Brainstem | 1.796(0.643, 5.019) | 0.264 |
| Ventricle oppressed | Yes | Ref |  |
|  | No | 1.399(0.848, 2.308) | 0.189 |
| En bloc resection | Yes | Ref |  |
|  | No | 0.598(0.354,1.009) | 0.054 |
| Ventricle opening | Yes | Ref |  |
|  | No | 1.247(0.732, 2.125) | 0.417 |
| Relapse | Yes | Ref |  |
|  | No | 0.498(0.292, 0.85) | 0.011 |
| KPS | >70 | Ref |  |
|  | ≤70 | 0.66(0.385, 1.132) | 0.131 |
| Pathological subtypes | A | Ref |  |
|  | AA | 0.41 (0.037, 4.593) | 0.469 |
|  | AO | 0(0, Inf) | 0.998 |
|  | AE | 0.421(0.038, 4.671) | 0.481 |
|  | O | 0(0, Inf) | 0.997 |
|  | GBM | 5.181(1.235, 21.73) | 0.025 |
| WHO Grade | Ⅱ | Ref |  |
|  | Ⅲ | 0.655(0.092, 4.665) | 0.672 |
|  | Ⅳ | 9.027(2.177,37.429) | 0.002 |
| Clinical Classification | LGG | Ref |  |
|  | HGG | 11.411(4.086, 31.867) | <0.0001 |
| Radio-Chemo therapy | No | Ref |  |
|  | Yes | 1.113(0.631, 1.964) | 0.71 |
|  | Single | 1.437(0.615, 3.358) | 0.402 |
| IDH mutation | Positive | Ref |  |
|  | Negative | 0.201(0.104, 0.391) | <0.0001 |
| MGMT methylation | Positive | Ref |  |
|  | Negative | 0.262(0.139, 0.495) | <0.0001 |
| 1P/19Q | No deletion | Ref |  |
|  | Co-deletion | 0.312(0.096, 1.011) | 0.052 |
|  | Single | 0.793(0.44, 1.429) | 0.44 |
| Ki67 | ≥30% | Ref |  |
|  | <30% | 1.26(0.763, 2.082) | 0.366 |
| A*β* | - | Ref |  |
|  | + | 0.537(0.308, 0.938) | 0.029 |

Number (n); Ventricle opening (Procedure in the tumor resection) Pathological pattern (A astrocytoma, O oligodendroglioma, AO anaplastic oligodendroglioma, AA anaplastic astrocytoma, AE anaplastic ependymoma, GBM glioblastoma); LGG (Lower-grade glioma, including WHO grade Ⅱ, Ⅲ); HGG (High-grade glioma, including WHO grade Ⅳ); A*β* status (- = negative stained, + = positive stained); Inf (Infinity).

**Table S3 Multi-variate Cox Regression analysis of risk factors for Overall Survival based on Survival status**

| **Characteristics** | **Classification** | **Hazard ratio (95% CI)** | ***p*-value** |
| --- | --- | --- | --- |
| Age | ≥45y | Ref |  |
|  | <45y | 3.816(1.821, 7.996) | <0.0001 |
| Gender | Male | Ref |  |
|  | Female | 1.069(0.532, 2.148) | 0.851 |
| Ventricle oppressed | Yes | Ref |  |
|  | No | 2.926(1.514, 5.656) | 0.001 |
| En bloc resection | Yes | Ref |  |
|  | No | 0.481(0.234, 0.988) | 0.046 |
| Relapse | Yes | Ref |  |
|  | No | 0.584(0.264, 1.293) | 0.185 |
| WHO Grade | Ⅱ | Ref |  |
|  | Ⅲ | 0.145(0.008, 2.692) | 0.195 |
|  | Ⅳ | 2.15(0.314, 14.746) | 0.436 |
| IDH mutation | Positive | Ref |  |
|  | Negative | 0.392(0.234, 0.988) | 0.048 |
| MGMT methylation | Positive | Ref |  |
|  | Negative | 0.435(0.201, 0.944) | 0.035 |
| 1P/19Q | No deletion | Ref |  |
|  | Co-deletion | 0.648(0.139, 3.029) | 0.582 |
|  | Single | 1.333(0.603, 2.95) | 0.478 |
| A*β* | - | Ref |  |
|  | + | 0.409 (0.181, 0.929) | 0.033 |

Number (n); Ventricle opening (Procedure in the tumor resection) Pathological pattern (A astrocytoma, O oligodendroglioma, AO anaplastic oligodendroglioma, AA anaplastic astrocytoma, AE anaplastic ependymoma, GBM glioblastoma); A*β* status (- = negative stained, + = positive stained); Inf (Infinity).

**Table S4** Experimental materials and reagents

| Name | Company | Species | Catalog |
| --- | --- | --- | --- |
| Anti- IBA-1 | Abcam | Rabbit | ab5076 |
| Anti- GFAP | DAKO | Mouse | Z033401-2 |
| Anti-CD3 | Biolegend | Rabbit | 100202 |
| Anti- CD68 | Biolegend | Rabbit | 137005 |
| Anti-IGF-1R | CST | Rabbit | 9750S |
| Anti- Phospho-IGF-1R | CST | Rabbit | 3024 |
| Ki-67 (D3B5) Rabbit mAb (Alexa Fluor® 647 Conjugate) | CST | Rabbit | 12075S |
| Alexa 488 Donkey Anti-Mouse IgG (H+L) | Thermo Fisher Scientific | Donkey | A21202 |
| Alexa-555 Donkey Anti-Rabbit IgG (H+L) | Thermo Fisher Scientific | Donkey | A21432 |
| IGFBP3 | Sigma | - | SRP3067 |
| CellTracke Red CMTPX | Yeasen | - | 40717ES50 |
| Green Actin Tracking Stain | Invitrogen | - | A57243 |

**Table S5 Primer sequences**

| Name | Forward primer sequence | | Reverse primer sequence |
| --- | --- | --- | --- |
| GAPDH | | 5'-CAGGTTGTCTCCTGCGACTT-3' | 5'-ATGTAGGCCATGAGGTCCAC-3' |
| IGF-1 | | 5'-GGCATTGTGGATGAGTGTTG-3' | 5'-TCTCCTTTGCAGCTTCGTTT-3' |
